# Nevus Lipomatosus Superficialis with Mixed Morphologic Features: Gross, Sonographic, and Histopathologic Correlation

**DOI:** 10.3390/life16040693

**Published:** 2026-04-21

**Authors:** Michelle T. Nguyen, Leo P. Wu, Grant M. Pham

**Affiliations:** 1 Naresh K. Vashist College of Medicine, Texas A&M University, College Station, TX 77843, USA; 2 Endeavor Health Elmhurst Hospital, Elmhurst, IL 60126, USA; 3 ExpertDerm Fellowship, Alpine, UT 84004, USA

**Keywords:** nevus lipomatosus superficialis, lipofibroma, point of care ultrasound, adipose disease, histopathologic correlation, case report

## Abstract

Nevus lipomatosus superficialis (NLS) is an uncommon benign hamartoma characterized by ectopic adipocytes within the dermis and may present with features that overlap clinically with other soft, pedunculated, or cerebriform lesions. We report a rare presentation with mixed morphologic traits that created diagnostic uncertainty on gross examination. The lesion demonstrated atypical surface contour and texture, prompting multimodal evaluation to clarify the differential diagnosis and support safe outpatient management. Point-of-care ultrasound (POCUS) was used to evaluate lesion architecture and vascularity. Findings provided real-time, noninvasive support for benign morphology and informed procedural planning. Subsequent histopathologic analysis established the diagnosis by demonstrating dermal adipose deposition consistent with NLS. This case underscores the value of integrating gross examination with sonographic assessment and histopathology when cutaneous lesions have overlapping clinical features. In addition, it contributes to the limited literature describing ultrasound findings in NLS. Incorporating POCUS into the assessment of atypical cutaneous growths may improve diagnostic confidence, reduce unnecessary escalation of care, and support efficient, safe treatment in outpatient settings.

## 1. Introduction

Nevus lipomatosus superficialis (NLS) is a rare, benign hamartomatous lesion characterized by mature adipocytes within the dermis [1]. Clinically, it is described in two principal phenotypes: a classical form that presents as a cerebriform plaque and a solitary form that presents as a pedunculated mass. The more common classical form tends to present earlier, often from birth through the third decade, whereas the solitary form more commonly presents in later adulthood, typically from the third to sixth decade [2,3]. Reported anatomic predilection also differs, with the classical form more often involving the pelvic girdle, trunk, and shoulder regions, while the solitary form more commonly involves the pelvic girdle and lower extremities [3,4]. Lesions that show overlapping features of both patterns appear to be uncommon and can create diagnostic uncertainty.

Sonographic characteristics of NLS are infrequently described in the literature, though previous descriptions of solitary NLS include an ovoid shape in the dermis to hypodermis with focal hypoechoic areas, posterior acoustic shadows, and a heterogenous texture [5]. The heterogenous texture correlates with the histopathological examination, as collagen fibers and adipose tissue are found to be woven together in varying degrees based on how much adipose tissue was present [4,6]. Clinically relevant mimickers include fibromas, giant acrochordons, lipomas, and neurofibromas [3,6]. Ultrasound can support differentiation, since lipomas more often localize to the hypodermis, while NLS characteristically involves the dermis. Furthermore, neurofibromas have a homogenous texture without a posterior acoustic shadow while NLS has a posterior acoustic shadow and is heterogenous [6]. Curative treatment for NLS is surgical resection of the lesion for cosmetic purposes, as they tend to not recur or be malignant [3]. The pathogenesis of NLS is unknown, though the leading theory by Hoffman and Zurhelle who first described NLS is that connective tissue degeneration leads to metaplastic adipose deposits in the dermis [2]. Other theories for formation of NLS include lipoblast differentiation into adipocytes from dermal pericytes and developmental disruption of adipose tissue forming a true nevus [1,5]. In this case report, we present a case of an atypical NLS with features of both the classical and solitary form.

## 2. Case Presentation

**Figure 1:**
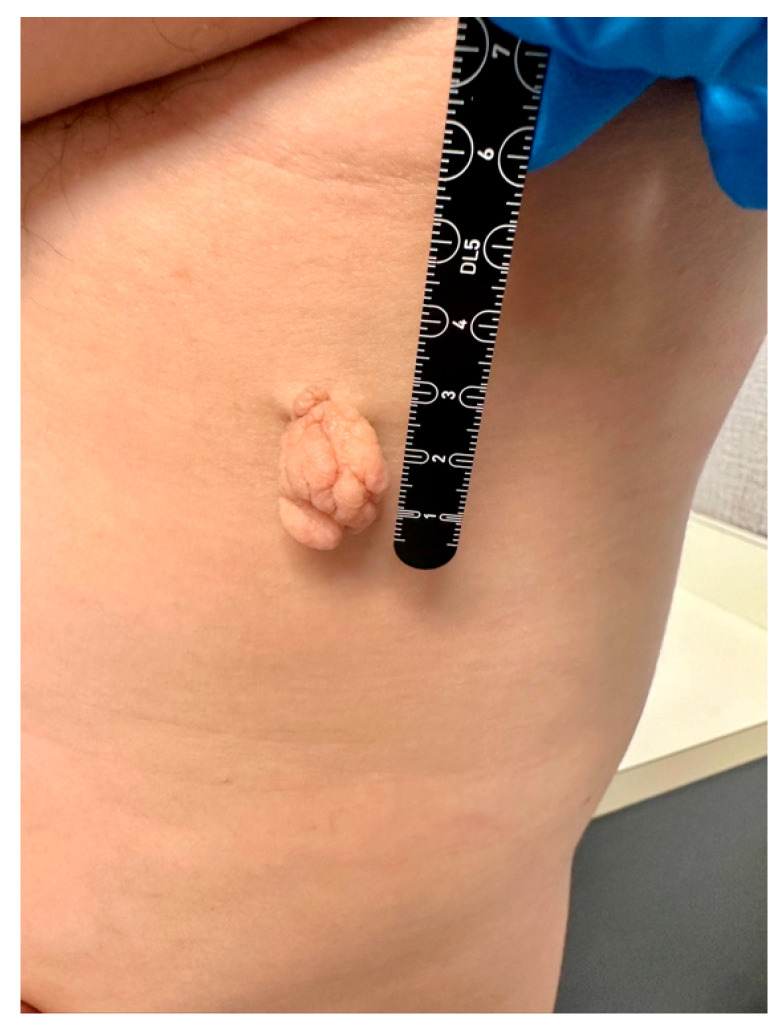
Solitary pedunculated cerebriform mass on the upper back.

**Figure 2:**
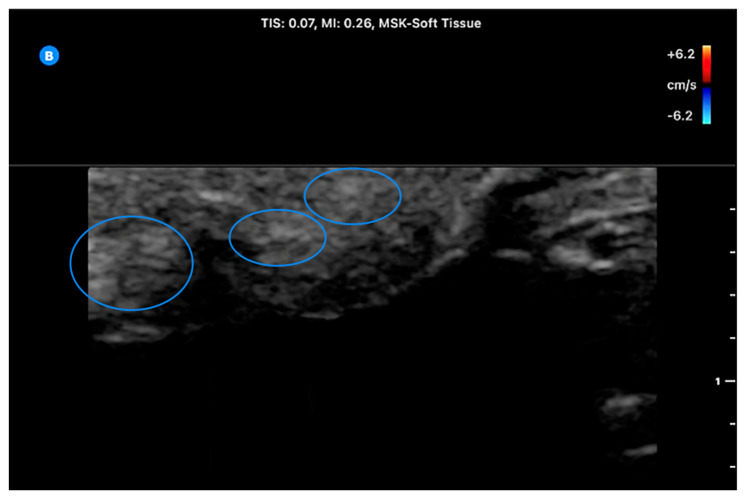
POCUS of the upper back lesion. Blue ovals represent hyperechoic foci likely a mix of soft tissue and adipose.

A 43-year-old male with Fitzpatrick type IV skin presented with a flesh-colored lesion on the upper back that had slowly enlarged over three years. He denied preceding trauma, similar prior lesions, and a non-pertinent family history. On examination, there was a lobulated pedunculated mass with a cerebriform surface pattern (Figure 1). The lesion measured approximately 2.1 × 2.3 × 1.7 cm^3^. It was soft, compressible, and freely mobile on its stalk. The surrounding skin was normal, and no regional lymphadenopathy was appreciated. Point-of-care ultrasound (POCUS) was performed using a Butterfly iQ3 handheld ultrasound system (Butterfly Network, Inc., Burlington, MA, USA). Imaging was obtained with a high-frequency linear preset for superficial soft tissue (MSK–Soft Tissue preset). Imaging demonstrated a superficially located mass with scattered hyperechoic foci suggestive of adipose content and no internal Doppler flow (Figure 2).

## 3. Management and Outcome

**Figure 3:**
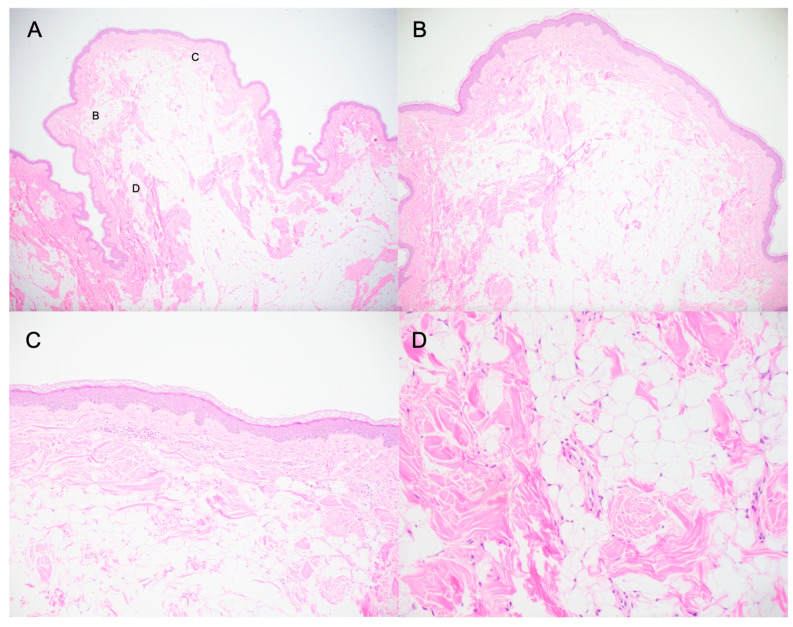
Histopathology of NLS stained with H&E at 2× (**A**), 4× (**B**), 10× (**C**), and 20× (**D**). In panel A, markers denote the regions corresponding to panels B, C, and D. On low power (panels **A**–**C**), there are aggregates of unencapsulated mature adipocytes within the superficial dermis. In some areas, the collagen bundles appear increased in density. On higher power (panel **D**), small-caliber blood vessels are present within the adipocytic aggregates with interspersed collagen bundles.

Given the lesion size and the discomfort it caused during daily activities, the patient elected to proceed with excision after shared decision making. The lesion was surgically excised and submitted for histopathologic evaluation. Hematoxylin and eosin (H&E) stained sections showed a polypoid skin lesion with a mildly acanthotic epidermis. The core was predominantly composed of mature adipose tissue with interspersed disorganized collagen bundles and small blood vessels. No cytologic atypia was identified, and the findings were consistent with NLS (Figure 3) [2]. Postoperative recovery was uneventful, and the patient remains recurrence free at three-month follow-up.

## 4. Discussion

As an uncommon hamartomatous lesion, NLS can present with variable clinical morphology. Our excised lesion showed cerebriform surface lobulation resembling the classical form, yet it also presented as a pedunculated mass consistent with the solitary variant. This mixed clinical pattern parallels the report by Lima et al. from 2017, in which the lesion combined features of both forms and histopathology confirmed the diagnosis [5]. In our case, the atypical morphology mimicked more common benign entities and complicated the clinical impression, making histopathologic confirmation essential.

The main clinical differential diagnoses included lipoma, giant acrochordon, and neurofibroma. On ultrasound, NLS has been described as an ill-defined hyperechoic dermal-based mass that may extend into the subcutis, often without internal Doppler flow, reflecting its adipose content [6,7]. In contrast, subcutaneous lipomas are typically well defined and ovoid with variable echogenicity and internal linear striations, whereas neurofibromas more often appear as well-defined hypoechoic solid masses and may demonstrate a target-like appearance [8]. Published sonographic descriptors for giant acrochordon are limited, but reported cases describe a superficial polypoid dermal lesion with a stalk and variable echogenicity depending on the degree of fibrous versus edematous stroma [9]. Comparative sonographic features are summarized in Table 1.

Beyond confirming diagnosis, this case invites consideration of how stromal, vascular, and immune components may shape the architecture of adipose-containing cutaneous hamartomas. Blood vessels are integral to cutaneous tissue organization, and immunohistochemical (IHC) methods can help further characterize vascular compartments in lesions that contain small-caliber vascular structures on routine histology [10]. Although lymphatic involvement has not been established in NLS, lymphatic biology has been linked to adipose tissue regulation and inflammatory remodeling in other settings, providing a potential framework for future investigation [11]. Immune populations involved in skin and adipose homeostasis, including group 2 innate lymphoid cells and macrophages, have also been associated with tissue remodeling and fibrosis [12,13]. While our evaluation was limited to H&E sections, future cases could consider targeted IHC characterization of vascular and immune components to further define the lesion microenvironment.

POCUS is increasingly used in outpatient settings because it is rapid, noninvasive, and does not involve ionizing radiation [14]. In this case, ultrasound contributed supportive information for a benign process and preprocedure assessment, although image quality was limited by the uneven cerebriform surface. This technical limitation is worth noting because irregular contact can introduce artifacts, and future reports may help define practical strategies to optimize imaging over lobulated lesions. A further limitation is that additional IHC staining was not performed because the tissue block was no longer available for further studies.

**Table 1:** Ultrasound patterns of case differentials.

Lesion	Typical Ultrasound Appearance
NLS	Reported as an ill-defined hyperechoic mass in the dermis or extending from dermis to subcutis (reflecting adipose content). Often no internal Doppler flow in described cases [6,7].
Lipoma (subcutaneous)	Classically well defined, often ovoid, with echogenicity that can be hyperechoic (common finding) but variable; may show linear internal striations. Usually minimal or no internal Doppler flow (benign) [8,14].
Neurofibroma	Often a well-defined hypoechoic solid mass, sometimes showing a target sign (hypoechoic rim with relatively echogenic center) in benign peripheral nerve sheath tumors. Variable; can show some Doppler flow [6,15].
Giant Acrochordon (skin tag, fibroepithelial polyp)	Direct published sonographic descriptors are sparse. Ultrasound may show a well-defined, heterogeneous hypoechoic mass with small anechoic areas. Color Doppler can demonstrate internal vascular signals in giant lesions if inflamed/irritated, supporting a benign but vascularized polypoid mass [9].

## 5. Conclusions

This case underscores the value of correlating gross examination, POCUS, and histopathology when evaluating cutaneous lesions with overlapping clinical appearances. In our patient, POCUS provided rapid, noninvasive support for a benign morphologic lesion and helped frame preprocedural assessment, while histopathology confirmed the diagnosis of NLS. These lesions with mixed classical and solitary features are rare, and sonographic descriptions remain limited. This case adds practical detail to multimodal evaluation of NLS. It also highlights a recognized diagnostic pitfall when clinical morphology overlaps with more common entities. Further work is needed to better define the pathogenesis of NLS, particularly in mixed presentations, and to clarify reproducible ultrasound characteristics that can aid outpatient diagnostic workflows.

## Data Availability

The original contributions presented in this study are included in the article. Further inquiries can be directed to the corresponding author.

## Abbreviations

The following abbreviations are used in this manuscript:

H&E	Hematoxylin and eosin
IHC	Immunohistochemical
NLS	Nevus lipomatosus superficialis
POCUS	Point-of-care ultrasound
